# Epicardial adipose tissue volume by cardiac magnetic resonance imaging predicts abnormal myocardial relaxation in patients with atrial fibrillation

**DOI:** 10.1186/1532-429X-17-S1-P352

**Published:** 2015-02-03

**Authors:** Rajeev R Fernando, Bassel Sayegh, Mushabbar A Syed, David Wilber, Sanjay Singh, Tonye Teme, Mark Rabbat

**Affiliations:** 1 Department of Internal Medicine, Loyola University Medical Center, Maywood, IL, USA; 2 Department of Cardiology, Loyola University Medical Center, Maywood, IL, USA; 3 Department of Biological Sciences, East-West University, Chicago, IL, USA

## Background

Inflammation may be a key trigger in the accumulation of extracellular matrix that leads to fibrosis, abnormal myocardial relaxation, diastolic dysfunction and eventual heart failure with a preserved ejection fraction (HFpEF). Epicardial adipose tissue (EAT) is a metabolically active organ releasing various adipokines and inflammatory mediators which may promote fibrosis in adjacent myocardium altering the structural properties of the ventricle leading to abnormal diastology. The aim of this study was to assess the relationship of EAT volume using cardiac magnetic resonance imaging (CMR) and parameters of diastolic dysfunction as assessed by 2D-transthoracic echocardiography (TTE) in patients with atrial fibrillation (AF).

## Methods

We conducted a retrospective study of 20 patients with AF who underwent CMR and TTE prior to ablation between 11/1/2010 and 10/17/2013 (Table 1). CMR Images were acquired on a 3T scanner (Siemens Trio) by steady state free precession (SSFP) using a standard short axis stack through the atria and ventricles. Epicardial adipose tissue quantification was peformed using a short axis stack through the atria and ventricles at end-diastole. For each short axis slice, the myo-epicardial and pericardial borders were manually traced and EAT volumes were calculated by summation of the area x thickness in each slice using CMR42 software (Circle CVI, Calgary, Alberta, Canada). For each TTE, pulsed wave Doppler of mitral inflow from the apical four chamber view and tissue Doppler of mitral annular excursion was performed. The following diastolic parameters were recorded for analysis: myocardial relaxation index (e' septal) and left ventricular filling pressures (E/e').

**Table 1:** Baseline patient characteristics

Parameters	Patients (n=20)
Age (years)	63.45+/-9.22

Male	12 (60)

Diabetes mellitus	1 (5)

Hypertension	10 (50)

Obstructive sleep apnea	1 (5)

Paroxysmal atrial fibrillation	15 (75)

Body mass index (kg/m2)	28.6 +/- 5

Body surface area (m2)	2.06 +/- 0.23

TTE: Myocardial relaxation index (septal e')	8.14 +/- 2.29

TTE: Left ventricular filling pressures (E/e')	10.75 +/- 3.13

CMR: Left atrial volume (mL)	104.5 +/-36.64

CMR: Left atrial volume index (mL/m2)	51.98 +/- 14.71

CMR: Epicardial adipose tissue volume (mL)	125.7 +/- 56.7

CMR:Left ventricular ejection fraction	60.93 +/- 4.81

Data are expressed as means +/- standard deviation or number (%) of patients

TTE: transthoracic echocardiography; CMR: cardiac magnetic resonance imaging

## Results

Patients with elevated filling pressures (E/e' >15) had significantly higher EAT compared to those with normal filling pressures (E/e' <15) (164ml±118 vs 114ml±54, p<0.001). By univariate analysis, EAT volume had a significant inverse correlation with e' sep (r =-0.48, p=0.02), but not hypertension (r=0.25, p=0.09), or E/e' (r = 0.22, p=0.21). EAT volume remained the only significant predictor of abnormal myocardial relaxation in a multivariate linear regression model incorporating age, BMI, LA volume, hypertension and coronary artery disease (p = 0.04).

## Conclusions

EAT volume quantified by CMR is independently associated with abnormal myocardial relaxation in patients with AF. EAT may serve as a novel therapeutic target to preserve diastolic function.

## Funding

None.

